# A Van Hiele–Newman model analysis of geometric reasoning and problem-solving errors among final-year students in a selected senior high school

**DOI:** 10.3389/fpsyg.2026.1870785

**Published:** 2026-10-12

**Authors:** Ebenezer Owusu, Yarhands Dissou Arthur, Ernest Larbi

**Affiliations:** Department of Mathematics Education, Akenten Appiah-Menka University of Skills Training and Entrepreneurial Development, Kumasi, Ghana

**Keywords:** generalized estimation equations, geometric reasoning, geometry achievement, Newman Error Hierarchical Model, problem-solving errors, Van Hiele levels

## Abstract

This study examined students’ geometric reasoning, geometric problem-solving errors, and the association between demonstrated Van Hiele reasoning levels and Newman error categories. A quantitative approach employing a cross-sectional descriptive-correlational design was adopted. The study involved 300 final-year students from a selected senior high school, who were selected through proportionate stratified random sampling. Data were collected using an adapted 20-item Geometry Test developed by Usiskin (1982) and a 12-item researcher-designed Geometry Problem-Solving Test aligned with the Ghanaian core mathematics curriculum. Descriptive statistics and category-specific binary logistic Generalized Estimating Equations (GEE) models were used for data analysis. Most students demonstrated lower levels of geometric reasoning, with Level 0 (visualization) and Level 1 (analysis) accounting for the largest proportions, while relatively few demonstrated Levels 2 (informal deduction) and 3 (deduction). Transformation errors were the most frequent, followed by comprehension errors, whereas reading errors were least frequent among the erroneous responses. The GEE analysis showed significant associations between demonstrated Van Hiele reasoning levels and comprehension, transformation, processing skill, and encoding errors, but not reading errors. The findings indicate that error patterns varied across demonstrated reasoning levels, highlighting the value of examining geometric problem-solving errors in relation to both the level of reasoning explicitly demonstrated and the stage at which difficulty was manifested. The study contributes an initial empirical examination of the proposed GRaPSE framework, which integrates these two dimensions for analyzing geometric problem-solving responses.

## 1 Introduction

Geometry remains a central strand of school mathematics because it supports logical reasoning, spatial visualization, and problem solving, while also serving practical functions in science, engineering, architecture, and everyday life (Mulligan, 2015; Sinclair and Bruce, 2015). Historically, geometry developed from human needs such as land measurement, construction, and design, and these practical origins continue to shape its educational relevance (Gray, 2007; Ostermann et al., 2012). In contemporary mathematics classrooms, students’ ability to identify geometric relationships, interpret figures, and justify solutions contributes not only to success in mathematics but also to broader analytical thinking and decision-making skills (Satır and Kurtuluş, 2024). For this reason, geometry occupies an important place in pre-tertiary mathematics curricula across many countries, including Ghana (Hourigan and Leavy, 2017; Akayuure, 2019; Akayuure et al., 2016).

Despite its importance, geometry continues to be one of the most difficult areas of mathematics for students (Adolphus, 2011). Several studies have reported persistent weaknesses in students’ understanding of geometric concepts, especially in areas involving visualization, deduction, proof, and application of theorems (Andira and Syam, 2022; Banson et al., 2022; Celik and Yilmaz, 2022). Evidence from the West African Senior School Certificate Examination (WASSCE) Chief Examiners’ Reports between 2012 and 2022 consistently points to poor student performance in geometry topics such as mensuration, construction, and circle theorems (Kpotosu et al., 2024). These reports attribute students’ low achievement to inadequate conceptual understanding, weak procedural knowledge, and difficulty interpreting problem requirements accurately. Similar observations by Maqoqa (2024) show that many learners struggle to connect related geometric ideas and often fail to visualize plane figures meaningfully, which affects their ability to select appropriate strategies and arrive at correct solutions.

Understanding these difficulties requires attention to how students reason geometrically (Mason, 2009; Sulistiowati et al., 2019). The Van Hiele model provides one of the most established frameworks for explaining students’ geometric thinking (Crowley, 1987; Fuys et al., 1988). The model proposes that students progress through hierarchical levels of reasoning: visualization, analysis, informal deduction, formal deduction, and rigor. Advancement from one level to the next depends on conceptual mastery rather than age alone, and students within the same classroom may operate at different reasoning levels (Senk, 1989; Van Hiele, 1986). Studies using this framework have shown that many secondary school students remain at lower levels of reasoning, which limits their ability to justify properties, make generalizations, and solve non-routine geometry problems (Bonyah and Larbi, 2021; Mawarsari and Dewi, 2023; Yalley et al., 2021).

While the Van Hiele model explains levels of geometric reasoning, it does not sufficiently identify the specific errors students make during problem solving. A student may be classified at a particular reasoning level, yet the exact point at which failure occurs in solving a geometry task may remain unclear. This makes error analysis an important complementary lens (Obeng et al., 2024). Newman’s Error Hierarchical Model (NEHM) addresses this need by identifying five stages where students may experience difficulty: reading, comprehension, transformation, processing skill, and encoding (Newman, 1977). In geometry, these errors may appear as misunderstanding geometric language, failure to interpret diagrams correctly, inability to select relevant theorems, procedural mistakes, or incorrect final expressions (Putri and Hastari, 2022; Obeng et al., 2024). Newman’s model, therefore, helps explain not only whether students fail, but how and where that failure occurs.

Existing studies have examined geometric reasoning levels and problem-solving errors largely as separate constructs (e.g., Naufal et al., 2021; Baffoe and Mereku, 2010). Many studies on the Van Hiele model focus on determining students’ reasoning levels without linking these stages to the specific difficulties evident in their problem-solving responses (e.g., Asemani et al., 2017; Bonyah and Larbi, 2021). Similarly, studies using Newman’s framework often identify error types without examining how these errors relate to the level of geometric reasoning demonstrated in the same problem-solving responses (e.g., Mawarsari and Dewi, 2023; Rahayu and Jupri, 2021). Research involving senior high school students in the Ghanaian context is also comparatively limited, as much of the literature has focused on junior high school learners or tertiary students. More importantly, the two frameworks have rarely been integrated at the level of individual problem-solving responses. Such integration is important because the Van Hiele model and Newman’s framework address different but complementary dimensions of problem-solving performance. The Van Hiele model identifies the level of geometric reasoning demonstrated, whereas Newman’s framework identifies the stage at which difficulty is manifested during problem solving. Examining these dimensions within the same response therefore provides a basis for determining whether particular Newman error categories are associated with different levels of demonstrated geometric reasoning.

This integration adds an analytical dimension that is not obtained by applying either framework independently. Rather than treating geometric reasoning level and problem-solving error as separate characteristics, the combined perspective allows an erroneous response to be examined simultaneously in terms of the highest level of geometric reasoning explicitly demonstrated and the Newman error category evident in that response. This provides a more differentiated account of geometric problem-solving difficulties by situating an observed error within the reasoning demonstrated in the response in which it occurred. The present study operationalizes this integration through the proposed Geometric Reasoning and Problem-Solving Errors (GRaPSE) framework, which brings the Van Hiele and Newman dimensions into a common analytical structure without assuming that one causes the other. The study therefore examines students’ geometric reasoning and problem-solving errors at both the student and response levels and investigates whether Newman error categories are associated with demonstrated Van Hiele reasoning levels. The study particularly focuses on final-year students in a selected senior high school, where empirical evidence on this integrated perspective remains limited. The investigation was guided by the following research questions:

- What are pre-tertiary students’ levels of geometric reasoning?
- What are students’ geometric problem-solving errors across demonstrated Van Hiele levels?
- Is there a significant association between demonstrated levels of geometric reasoning and problem-solving errors?

## 2 Literature review

### 2.1 Students’ geometric reasoning across Van Hiele levels

Research on students’ geometric reasoning consistently shows that many students operate at lower Van Hiele levels, even when curriculum expectations require higher-order deductive thinking (Ngirishi and Bansilal, 2019; Priliawati et al., 2019). The Van Hiele model explains geometric reasoning as a hierarchical progression from visualization to rigor, where students move from recognizing shapes by appearance to engaging in formal proof and abstract reasoning (Van Hiele, 1986). Across educational contexts, studies have repeatedly shown that progression beyond the first two levels remains limited, creating challenges for geometry learning and the development of more advanced geometric reasoning (e.g., Alex and Mammen, 2012; Asemani et al., 2017; Naufal et al., 2021). Students at the visualization level often identify shapes based only on appearance and struggle when figures are rotated or presented in unfamiliar forms. At the analysis level, learners can describe properties of shapes but often fail to establish relationships among those properties. As a result, tasks involving proof, justification, and theorem application may become difficult. Andira and Syam (2022) found that students at lower Van Hiele levels were unable to progress through the stages of problem solving proposed by Pólya (1945), while those at higher levels demonstrated stronger strategic reasoning and solution planning. Similarly, Satır and Kurtuluş (2024) observed that misconceptions about quadrilaterals were more common among students operating at visualization and analysis levels, whereas students at the informal deduction level showed fewer conceptual errors.

A common finding in the literature is that most students are concentrated at the visualization and analysis levels, with very few reaching informal deduction or formal deduction. For example, Alex and Mammen, 2012 found that most Grade 10 students remained at Levels 0 and 1, while only a small proportion demonstrated Level 3 reasoning. Similar patterns were reported by Ramadhania et al. (2022), where more than half of the students were at the visualization level, and only a few reached informal deduction. Asemani et al. (2017) also reported that a substantial proportion of final-year senior high school students could not attain any identifiable Van Hiele level, with less than 3% reaching formal deductive levels. The persistence of low reasoning levels is not limited to school students alone. Studies involving pre-service teachers report similar concerns. Bonyah and Larbi (2021), as well as Salifu et al. (2018), found that elementary pre-service mathematics teachers in Ghana demonstrated weak geometric thinking across the first three Van Hiele levels. This raises concerns for classroom instruction because teachers with limited geometric reasoning may struggle to guide students toward higher-order thinking.

Although the literature strongly establishes that many students remain at lower Van Hiele levels, most studies focus primarily on identifying reasoning stages without examining how these levels relate to the specific errors students make during problem solving. Many studies stop at classifying students’ geometric thinking without extending the analysis to how those reasoning levels are associated with problem-solving. This limits practical intervention because knowing that a student is at Level 1 does not fully explain where failure occurs during task completion. A more useful understanding requires linking reasoning levels to the nature of students’ problem-solving errors.

### 2.2 Students’ geometric reasoning level and problem-solving errors

Geometry requires students to interpret diagrams, identify properties and relationships, apply geometric principles, and communicate solutions logically (Kania et al., 2024). Difficulties can therefore arise at different stages of the problem-solving process, making error analysis an important approach for identifying where students experience difficulty and for understanding the nature of their responses (Widiawati et al., 2020; Odor et al., 2024; Repriani and Jupri, 2025).

Newman’s Error Analysis provides a useful framework for examining these difficulties by locating errors at different stages of the problem-solving process (Newman, 1977). Research using the framework has identified comprehension, transformation, processing skill, and encoding errors among the recurring errors in geometry problem solving (Odor et al., 2024; Repriani and Jupri, 2025). Widiawati et al. (2020) and Kania et al. (2024), for example, reported errors in transforming verbal information into appropriate mathematical representations or diagrams, even when students were able to identify relevant information from the problem. Similarly, Elsa et al. (2022) reported conceptual and representational errors in solid geometry, particularly when students were required to connect verbal descriptions with spatial representations. Transformation errors may be particularly important in geometry because solving many geometric problems requires movement between verbal, symbolic, and visual representations (Widiawati et al., 2020; Kania et al., 2024; Nuritasari and Aini, 2023).

Errors may also occur at later stages of problem solving. Riastuti et al. (2017) and Dewi et al. (2020) found that students with weaker spatial abilities experienced difficulties in transforming geometric information and carrying out appropriate procedures. Zamzam and Patricia (2018) similarly reported difficulties in selecting suitable operations and connecting diagrams with formal procedures. Processing skill and encoding difficulties have also been observed in tasks requiring formal procedures and proof. Azkiana et al. (2025) found that students could identify an appropriate theorem or formula but still encounter difficulties during its execution or in presenting the final answer, while Shinariko et al. (2020) showed that even high-achieving students could experience procedural and communication errors. These findings indicate that geometry errors can occur at different stages of problem solving and are not necessarily confined to students who demonstrate generally weak performance.

The nature of these errors also needs to be considered in relation to students’ geometric reasoning. The Van Hiele model provides a framework for understanding differences in how students conceptualize geometric objects, properties, and relationships. Students operating at lower levels of geometric reasoning may rely more heavily on the visual appearance of figures or on isolated properties, whereas students at higher levels are increasingly able to coordinate properties and reason about their relationships (Miatun et al., 2021; Putri and Nurlaelah, 2022). Consequently, the reasoning level demonstrated by a student may be relevant to the kinds of errors that become visible during problem solving. For example, students functioning at the visualization level may have difficulty recognizing relevant geometric properties when figures are presented in unfamiliar forms, while students at the analysis level may identify individual properties without establishing relationships among them (Mosia et al., 2023; Satır and Kurtuluş, 2024). Such difficulties may be manifested as comprehension or transformation errors when students are required to interpret problem conditions, construct representations, or select an appropriate solution strategy (Dewi et al., 2020; Putri and Nurlaelah, 2022). At higher reasoning levels, students are better positioned to establish relationships among geometric properties and use logical connections in solving problems (Celik and Yilmaz, 2022). However, higher reasoning does not necessarily eliminate problem-solving errors. Students may understand the relevant geometric relationships but still experience difficulties when coordinating several properties, applying procedures, constructing formal arguments, or communicating a solution (Miatun et al., 2021; Mosia et al., 2023). This suggests that the relationship between geometric reasoning and problem-solving errors may be more differentiated than a simple progression from more errors at lower levels to fewer errors at higher levels. The particular stage at which an error occurs may vary according to the reasoning demonstrated in the response and the demands of the problem being solved.

Empirical studies provide some evidence for this relationship between Van Hiele reasoning and error patterns. Dewi et al. (2020) reported that students at lower Van Hiele levels demonstrated a wider range of Newman error types than students at higher levels. Similarly, Putri and Nurlaelah (2022) observed that students’ error patterns became less varied and more specific as their geometric reasoning developed. Mosia et al. (2023) found that many errors occurred among students functioning at the visualization and analysis levels, even when the tasks required more advanced deductive reasoning. Mawarsari et al. (2023) likewise reported that students at the visualization level demonstrated all five Newman error types, whereas students at higher reasoning levels exhibited fewer and more specific error types. Taken together, these studies indicate that the types and distribution of observable problem-solving errors can vary across levels of geometric reasoning.

However, the existing literature does not yet provide a sufficiently detailed account of how specific Newman error categories correspond to the Van Hiele reasoning demonstrated within individual problem-solving responses. Although some studies have considered Van Hiele reasoning and Newman error analysis together (Dewi et al., 2020; Putri and Nurlaelah, 2022), the evidence has largely been used to describe or compare error patterns across reasoning levels. This leaves a more specific analytical question concerning whether the Van Hiele level explicitly demonstrated in an individual erroneous response is associated with the type of Newman error manifested in that same response. Such response-level analysis is important because a student may demonstrate different levels of geometric reasoning across different problems, and a student-level Van Hiele classification may therefore not adequately represent the reasoning demonstrated in every problem-solving response. The present study addresses this gap by bringing the two analytical perspectives together at the level of the individual erroneous response. This approach makes it possible to examine the correspondence between demonstrated geometric reasoning and specific problem-solving error categories rather than treating reasoning level and error type as separate descriptions.

### 2.3 Theoretical framework

#### 2.3.1 The Van Hiele model of geometric reasoning

This study was anchored in the Van Hiele Model of Geometric Reasoning, which served as a diagnostic and interpretive framework for assessing students’ levels of geometric reasoning. The model remains one of the most widely accepted frameworks in mathematics education research for explaining how students develop geometric understanding and how they reason when solving geometry-related tasks (Bonyah and Larbi, 2021; Sulistiowati et al., 2019; Salifu et al., 2018; Asemani et al., 2017). The Van Hiele model proposes that students progress through hierarchical levels of geometric reasoning, with advancement depending on mastery of the preceding level rather than age alone (Van Hiele, 1986). This means that progression is not automatic, as a student cannot meaningfully function at a higher level without first developing understanding at the preceding level (Fuys et al., 1988). The five levels are visualization, analysis, informal deduction, formal deduction, and rigor. Evidence from studies involving senior high school students suggests that geometric reasoning at this level commonly spans the first four levels, namely, visualization, analysis, informal deduction, and formal deduction, while attainment of the fifth level, rigor, is less commonly reported (Asemani et al., 2017; Mudhefi et al., 2024; Usiskin, 1982). Accordingly, the present study focused on the first four levels in assessing students’ overall geometric reasoning.

At Level 0, visualization, students recognize geometric figures based on their overall appearance rather than their defining properties (Kekana, 2016). Thinking at this level is largely perceptual, and recognition depends on familiar prototypes (Bonyah and Larbi, 2021). Level 1 (analysis) is characterized by students’ ability to identify and describe the properties of shapes (Alex and Mammen, 2016). Learners begin to move beyond appearance and focus on attributes such as equal sides, angles, and parallel lines. They can list these properties, but they often fail to recognize relationships among them. At Level 2 (informal deduction), students begin to connect the properties of geometric figures and use informal logical reasoning to organize and classify shapes based on these relationships (Celik and Yilmaz, 2022). Moving to Level 3 (deduction), students progress beyond accepting given evidence and become capable of constructing proofs independently, using logical arguments to justify geometric theorems (Arnal-Bailera and Manero, 2024). Students’ understanding and approaches to solving geometric problems differ from one level to another, given that each stage is characterized by specific benchmarks.

#### 2.3.2 The Newman error hierarchical model

This study was also guided by the Newman Error Hierarchical Model (NEHM), which served as a diagnostic framework for identifying the specific stages at which students experience difficulties when solving geometry problems. Newman (1977) argued that students’ errors in mathematics problem solving do not occur randomly but arise systematically at identifiable stages of cognitive processing. The model has been widely used in mathematics education research to examine students’ mistakes and explain the underlying causes of failure in problem solving (Arhin and Hokor, 2021; Kristianti and Retnawati, 2020; Obeng et al., 2024).

The Newman model proposes that successful problem solving requires students to pass through five hierarchical stages: reading, comprehension, transformation, processing skill, and encoding. Failure at any stage affects progress at subsequent stages and may prevent students from arriving at the correct solution (Newman, 1977). Reading errors (RE) occur when students are unable to correctly recognize mathematical words, symbols, or notations in a problem (Alhassora et al., 2017). Comprehension errors (CE) arise when students can read the problem but fail to understand what is required (Zamzam and Patricia, 2018). Transformation errors (TE) occur when students understand the question but cannot translate it into an appropriate mathematical or geometric representation (Riastuti et al. (2017)). Processing skills errors (PSE) involve mistakes in carrying out the required procedures or calculations, even when the correct method is known (Obeng et al., 2024). Encoding errors (EE) occur when students fail to present the final answer correctly using the appropriate notation, unit, or format (Dewi et al., 2020). In the context of the present study, the Newman model provided a useful lens for examining the geometric errors committed by SHS students. While the Van Hiele model explains students’ levels of geometric reasoning, the Newman model helps identify where and why students experience difficulty during problem solving. This makes the two frameworks complementary, as Van Hiele explains the development of geometric thinking, whereas Newman explains the cognitive obstacles that hinder that development.

### 2.4 Conceptual framework

Drawing on the Van Hiele model of geometric reasoning and the Newman Error Hierarchical Model, this study proposes an integrative framework termed “GRaPSE” (Geometric Reasoning and Problem-Solving Errors). The framework is proposed as an analytical lens for examining two dimensions of students’ geometric problem-solving responses that are addressed separately by the two established models: the level of geometric reasoning demonstrated in a response and the stage of problem solving at which an error occurs. The theoretical rationale for their integration is that an error observed in a geometric problem-solving response may be interpreted more meaningfully when considered alongside the level of geometric reasoning demonstrated in that same response. The two models make distinct but complementary contributions to this interpretation. The Van Hiele model provides a developmental account of geometric reasoning, distinguishing progressively more sophisticated ways in which learners recognize, analyze, relate, and formally reason about geometric concepts. The Newman Error Hierarchical Model, in contrast, provides a diagnostic account of problem-solving difficulties by locating errors at different stages, including reading, comprehension, transformation, processing skill, and encoding. Thus, while the Van Hiele model addresses the nature and level of reasoning demonstrated, the Newman model addresses the stage at which difficulty is manifested during problem solving. GRaPSE brings these dimensions into a common analytical structure rather than treating them as independent descriptions of students’ responses.

The distinctive proposition of GRaPSE is therefore not simply the simultaneous use of the two models, but their integration at the level of individual problem-solving responses. Within this framework, each response can be examined according to the highest Van Hiele level of reasoning explicitly demonstrated and, where the response is erroneous, the corresponding Newman error category identified in that same response. This creates a two-dimensional representation of an erroneous response: the level of geometric reasoning demonstrated and the stage of problem-solving difficulty observed. This integration therefore shifts the analysis from identifying either students’ geometric reasoning levels or their problem-solving errors independently to examining how the two dimensions are represented together within the same problem-solving response. GRaPSE also distinguishes between overall student-level geometric reasoning and response-level demonstrated reasoning.

Accordingly, GRaPSE conceptualizes an individual geometric problem-solving response through the intersection of two analytically distinct dimensions: (a) the level of geometric reasoning demonstrated and (b) the stage of problem-solving difficulty represented by a Newman error category. The framework does not posit that a particular Van Hiele level causes a particular type of error. Rather, it provides a structure for examining whether patterns of co-occurrence exist between demonstrated reasoning levels and error categories in students’ geometric problem-solving responses. It is noteworthy that GRaPSE is proposed in this study as an integrative analytical framework rather than as an established or empirically validated theory. The present study provides an initial empirical examination of the associations between response-level demonstrated Van Hiele reasoning and Newman error categories within the study context. The observed associations should therefore be interpreted as empirical evidence relevant to the proposed framework, rather than as validation of the framework itself. Further research using different samples, contexts, geometric tasks, and research designs is needed to examine the robustness, explanatory utility, and empirical validity of GRaPSE.

Figure 1 presents the proposed GRaPSE framework. The Van Hiele dimension represents progressive levels of geometric reasoning, while the Newman dimension represents stages at which difficulties may be manifested during problem solving. Their intersection represents the analytical space in which an individual problem-solving response can be examined in terms of both the reasoning demonstrated and the type of error observed. The framework therefore provides the conceptual basis for examining patterns of association between these two dimensions without assuming a causal or directional relationship between them.

**Figure 1:**
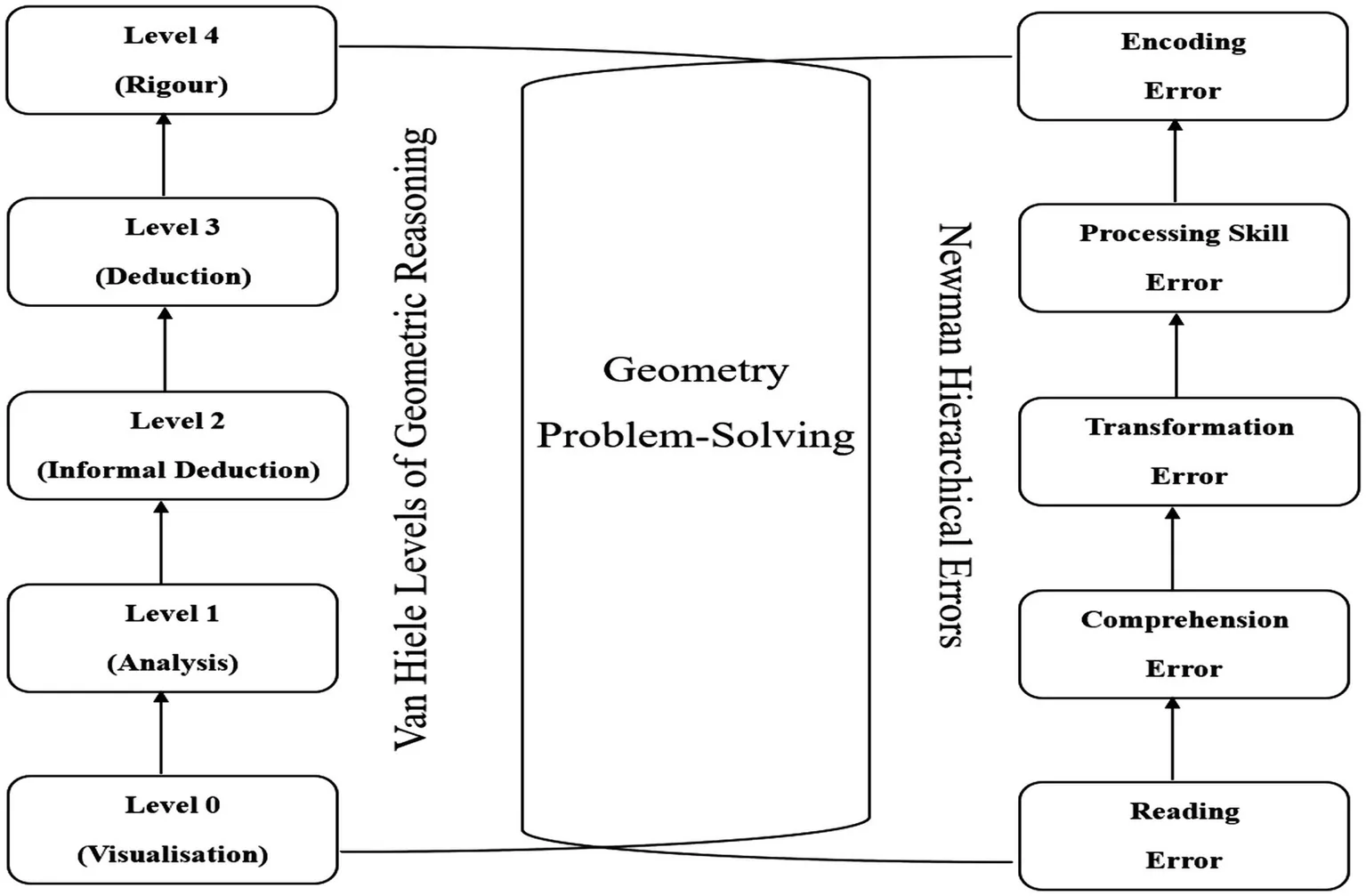
Conceptual Framework (The GRaPSE Model). Source: Authors’ Construct.

## 3 Methodology

### 3.1 Research approach and design

This study focused on analyzing students’ geometric reasoning levels and geometric problem-solving errors using the Van Hiele model of geometric reasoning and the Newman Error Hierarchical Model. Since the study sought to examine the association between demonstrated geometric reasoning levels and problem-solving error categories using data collected from respondents at a single point in time, without manipulating the variables or introducing any intervention, a quantitative approach was considered appropriate. In addition, the findings were reported descriptively while the association between the variables was examined statistically. Therefore, the study adopted a cross-sectional descriptive-correlational design (Sokratis et al., 2017; Sokratous et al., 2013; Sharifi et al., 2020).

### 3.2 Population, sample and sampling techniques

The target population for this study comprised all final-year pre-tertiary students in Ghana. However, due to practical constraints, a selected Senior High School constituted the accessible population of 990 students drawn from different academic programs such as General Arts, General Science, Business, Home Economics, and Visual Arts. Final-year students were considered appropriate for the study because they had received sufficient exposure to geometry instruction at the pre-tertiary level and were therefore better positioned to demonstrate their geometric reasoning levels and problem-solving abilities. The sample size for the study was determined using an online sample size calculator^1^, which automatically computes the minimum sample size. Given the population size (990), confidence level (95%), margin of error (5%) and estimated proportion (50%), the computed sample sizes were 285, 277, and 277 for Yamane (1967), Cochran (1977) and Krejcie and Morgan (1970), respectively. To account for possible non-response, incomplete scripts, and to improve representation across the various academic programs, the sample size was increased to 300 students. A stratified random sampling technique was employed for participant selection. The population was first divided into strata based on students’ academic programs of study. After this, proportionate stratified random sampling was used to ensure that each program was represented according to its actual population size. Within each stratum, students were selected randomly, giving every final-year student an equal chance of being included in the study.

### 3.3 Data collection instrument

The assessment of students’ overall geometric reasoning levels was conducted using an adapted version of the Van Hiele Geometry Test (VHGT) developed by Usiskin (1982). Prior to the use of the instrument, formal permission was sought from the developer, Professor Zalman Usiskin, through email, and approval was granted for its use in the present study. The original VHGT has been widely used to diagnose students’ geometric thinking levels (Chen et al., 2019; Haviger and Vojkůvková, 2015; Kim, 2016). For the present study, the instrument was restricted to 20 multiple-choice items covering Levels 0 to 3, namely visualization, analysis, informal deduction, and deduction. This decision was guided by evidence that geometric reasoning among senior high school students commonly spans these first four levels, while attainment of the fifth level is relatively uncommon in this population (Asemani et al., 2017; Bonyah and Larbi, 2021; Baffoe and Mireku, 2010; Mawarsari and Dewi, 2023; Yalley et al., 2021). The adapted form therefore retained five items for each of the four assessed levels, providing representation of each level while keeping the assessment focused on the range of reasoning levels most relevant to the study population. Each level consisted of five items requiring students to demonstrate the specific forms of reasoning in (𝑛 − 1) level before proceeding to the next level (𝑛) by answering at least three of the five items associated with that level correctly, consistent with the level-classification procedure adopted in the study. Because the present study used a shortened form of the original VHGT and did not assess Level 4 (rigor), the resulting classification represents students’ demonstrated reasoning within Levels 0 to 3 rather than a complete assessment of the full five-level Van Hiele hierarchy. Accordingly, evidence concerning the validity of the original VHGT was used to support the theoretical and measurement basis of the instrument, while the present adaptation was interpreted within the specific scope and population of the study. The responses to the VHGT were used solely to classify each student’s overall Van Hiele reasoning level.

To identify students’ geometric problem-solving errors and the Van Hiele reasoning demonstrated within individual problem-solving responses and for the purpose of answering RQ2 and RQ3, a 12-item researcher-designed Geometry Problem-Solving Test (GPST) (see Appendix B) was developed. The test was aligned with the Ghanaian Core Mathematics Curriculum, particularly Strand 3 (Geometry Around Us), and was informed by WASSCE past questions to reflect authentic examination demands for final-year SHS students. Most of these questions were WASSCE past questions; however, reframe in a non-routine or problem-solving format. The items focused on spatial reasoning, diagram interpretation, measurement, and geometric application skills. All questions required open-ended written responses, which made it possible to examine each response for the highest demonstrated Van Hiele reasoning level and, where an error occurred, classify the corresponding error using the Newman Error Hierarchical Model.

Because the GPST items differed in their mathematical content and the opportunities they provided for demonstrating geometric reasoning, Van Hiele levels were not assigned *a priori* to individual items. Instead, the level recorded for each response represented the highest level of geometric reasoning explicitly demonstrated in the student’s written work. This approach distinguishes the reasoning potential of a task from the reasoning actually evidenced in a response. Thus, a lower level assigned to a response does not imply that the student possessed only that level of geometric reasoning; it indicates the highest level that could be identified from that particular response. The response-level classification was therefore used to characterize demonstrated reasoning within individual problem-solving episodes rather than to reclassify students’ general Van Hiele levels.

### 3.4 Validity and reliability measures of the instrument

The VHGT developed by Usiskin (1982) was used to assess students’ geometric reasoning levels. The instrument has been widely validated in mathematics education research and has consistently demonstrated strong construct validity in measuring hierarchical levels of geometric reasoning across different educational contexts (Chen et al., 2019; Kim, 2016; Mayberry, 1983). For the researcher-designed Geometry Problem-Solving Test (GPST), content validity was ensured through alignment with the Ghanaian Core Mathematics Curriculum, WASSCE test formats, and the expected geometric competencies of final-year SHS students. In addition to this, the draft items were further subjected to expert review by two mathematics education specialists who examined the instrument for clarity, curriculum relevance, cognitive suitability, and alignment with the Newman Error Hierarchical Model. Using Gregory’s (2007) content validity approach, a validity coefficient of 0.83 was obtained, indicating strong content validity. Necessary revisions were made based on the experts’ suggestions.

Reliability of the instruments was established through pilot testing involving 30 students, at the same level as the study’s participants; however, these students were not included in the main study. All reliability coefficients were calculated using the Statistical Package for the Social Sciences (SPSS, Version 27). For the Van Hiele Geometry Test, internal consistency was assessed using Cronbach’s alpha, which yielded a coefficient of 0.78, indicating acceptable reliability. Similarly, the GPST (see Appendix A) recorded a split-half coefficient of 0.73, indicating acceptable consistency across the items used to assess students’ responses to the geometric problem-solving tasks. Since the GPST also involved coding responses according to the demonstrated Van Hiele reasoning level and classifying errors based on the Newman Error Hierarchical Model, inter-rater reliability was assessed using Cohen’s kappa to ensure consistency in coding. Two raters with expertise in mathematics education and students’ mathematical error analysis independently examined the relevant student responses using the established coding criteria for both demonstrated Van Hiele reasoning levels (see Appendix C) and Newman error categories (see Appendix E). For each response, the raters identified the highest level of geometric reasoning explicitly demonstrated. Where a response was erroneous, they also identified the corresponding Newman error category based on the stage of problem solving at which the difficulty was manifested. Cohen’s kappa coefficient (0.79) was calculated from the raters’ initial independent coding decisions to assess inter-rater agreement. The number of scripts included in this analysis was 30. Following this assessment, the raters compared their classifications and reviewed disagreements with reference to the operational definitions in the coding manual. Disagreements concerning either the demonstrated Van Hiele reasoning level or the Newman error category were resolved through discussion and consensus. The agreed classifications were subsequently used in the final dataset.

### 3.5 Data collection and analysis procedures

Prior to data collection, ethical approval was obtained from the Institutional Review Board of the University of Skills Training and Entrepreneurial Development (USTED), after which permission was sought from the authorities of the selected School. Students were briefed on the purpose of the study, confidentiality of responses was assured, and participation was made voluntary. Unique identification numbers were assigned to students to ensure anonymity and consistency during scoring and analysis. Data collection was carried out under normal classroom examination conditions. The Van Hiele Geometry Test was administered first and lasted 45 min, followed by a 30-min break and the administration of the researcher-designed GPST, which lasted 1 h. Given that the written responses were needed for both scoring and error analysis, students were reminded to explicitly show their workings so that their responses could be examined for both problem-solving errors and demonstrated geometric reasoning. Students completed both tests individually without external assistance, and all scripts were collected, coded, and securely stored for analysis. Consistent with studies (e.g., Asemani et al., 2017; Baffoe and Mereku, 2010; Salifu et al., 2018), responses from the VHGT were scored according to the procedure proposed by Usiskin (1982), with one mark awarded for each correct response across the 20 multiple-choice items. Students’ geometric reasoning levels were determined using the “3 out of 5” criterion for each level and the modified weighted scoring system recommended by Usiskin (1982), where Levels 0 to 3 were assigned a weighted sum of 1, 2, 4, and 8, respectively. This approach was adopted because it provides a more consistent classification of students’ reasoning levels (Usiskin, 1982).

Students’ responses to the GPST were scored using a standardized marking guide developed during the pilot stage. Following this, each written response was examined separately to identify the highest Van Hiele reasoning level demonstrated in that response. The coding was based on the reasoning explicitly evidenced in the student’s written work rather than on a Van Hiele level assigned *a priori* to the GPST item. This approach recognized that the GPST items differed in content and in the opportunities they provided for demonstrating higher-level geometric reasoning. A response was therefore assigned a particular Van Hiele level only when evidence of reasoning corresponding to that level was present in the written response. The resulting classification represented the highest level demonstrated within that response and was not interpreted as the student’s general or stable Van Hiele reasoning level. The student-level VHGT classification, including the “No identifiable level” category, was not transferred or used to assign Van Hiele levels to individual GPST responses. Thus, students classified as having “No identifiable level” on the VHGT were not excluded from the GPST analysis on that basis. Their individual GPST responses were independently coded according to the highest Van Hiele reasoning level demonstrated in each response. Because the VHGT classification and the GPST response-level coding served different analytical purposes and used different units of analysis, the distribution of students across VHGT levels was not expected to correspond numerically to the distribution of GPST responses across demonstrated Van Hiele levels. For incorrect responses, the Newman Error Hierarchical Model was then used to identify and classify the error as RE, CE, TE, PSE, or EE. The demonstrated Van Hiele level and Newman error category were linked within the same erroneous response for subsequent analysis. Correct and unattempted responses were excluded from the error-level analysis. Thus, for RQ2 and RQ3, the unit of analysis was the individual erroneous GPST response rather than the student. The 2,168 erroneous responses were contributed by the 300 students, with individual students contributing multiple responses across the 12 GPST items. The detailed response-level coding and analysis procedure is provided in Appendices C–E.

Descriptive statistics were used to summarize students’ overall Van Hiele reasoning levels and the distribution of Newman error categories across demonstrated reasoning levels. Given the repeated observations contributed by students, and consistent with the use of GEE for analyzing correlated observations (Lipsitz et al., 1994; Wilson and Lorenz, 2015; Wilson et al., 2024), five separate binary logistic Generalized Estimating Equations (GEE) models were fitted for the inferential analysis, with each Newman error category coded as 1 and all other error categories coded as 0. Student identification (ID) number was specified as the subject variable to account for the repeated observations contributed by students, while GPST Item was included as a categorical explanatory factor in the models to account for differences among the test items, including differences in content and cognitive demand, and as the within-subject repeated-measures factor to account for the repeated responses across the test items. An exchangeable working correlation structure was specified. Demonstrated Van Hiele reasoning level was entered as a categorical predictor, with Level 3 serving as the reference category. Holm adjustment was applied across the five omnibus tests to control the family-wise error rate. The analysis therefore examined whether the odds of a particular Newman error category, relative to the other error categories, differed according to the Van Hiele reasoning level demonstrated within erroneous responses. Statistical significance was evaluated at the 0.05 level.

### 3.6 Ethics declaration

The study was approved by the Institutional Review Board (IRB) of the University of Skills Training and Entrepreneurial Development (USTED) with reference number USTED/IERC/2026/089 under a Master of Philosophy in Mathematics Education Thesis (Protocol) Title: “Analysis of pre-tertiary students’ geometric reasoning and problem-solving errors: a van Hiele-Newman model perspective.” The study adhered strictly to the principles outlined in the Declaration of Helsinki (World Medical Association, 2013). Participation was voluntary, informed consent, confidentiality, and the right to withdraw without penalty were all ensured. The purpose of the study was shared with participants. For students below 18 years, parental or guardian consent was obtained alongside student assent. Anonymity was ensured through the use of identification codes, and no personal identifiers were included in the dataset or reports. Data were securely stored and accessed only by the researcher. Approval was also obtained from the developer of the Van Hiele Geometry Test prior to its use.

## 4 Results

### 4.1 Profile of participants

The study comprised 300 final-year (Form 3) students. The sample included 150 males (50.0%) and 150 females (50.0%). Regarding age, 45 students (15.0%) were between 15 and 17 years, 235 (78.3%) were between 18 and 20 years, and 20 (6.7%) were aged 21 years or older. All participants were in Form 3 at the time of the study. The demographic characteristics of the participants are presented in Table 1.

**Table 1:** Profile of participants.

Demographics	Frequency (N)	Percentages (%)
Gender	300	100.0
Male	150	50
Female	150	50
Age	300	100.0
15–17 years	45	15.0
18–20 years	235	78.3
>21	20	6.7
Form	300	100.0
Form 3	300	100

Values in bold denote total frequency (*N* = 300) and percentages (100%).

### 4.2 Students’ Van Hiele geometric reasoning levels

#### 4.2.1 Descriptive statistics of scores obtained by students in the VHGT

As shown in Table 2, there was variability in the geometric reasoning scores among the study’s participants. For a score range of 2 to 13, the majority (*N* = 185, 61.7%) of the students achieved scores between 5 and 8 marks, suggesting that most students’ VHGT scores were concentrated within this range. Lower scores were less frequent, with only 11 students (3.7%) obtaining a score of 2 and 18 students (6.0%) scoring 3 out of the total possible 20 marks. This is an indication that, in terms of test performance, a small number of students demonstrated minimal reasoning. Likewise, those achieving higher scores were found to be few, with only 5 students (1.7%) scoring 12 or 13. It is clear from the table that, out of the 300 participants, 267 students, accounting 89%, earned a score below the average mark of 10, demonstrating students’ limited mastery of geometric concepts assessed by the test. In spite of all this low performance, no mark below 2 was recorded, and the result suggests that at least all students possess some level of geometric reasoning. Presented in Table 3 are the descriptive statistics of the participants’ general performance in the VHGT.

**Table 2:** Overall scores achieved in the VHGT items.

Scores	Number of students (*n*)	Cumulative (*n*)	Percentage (%)	Cumulative (%)
2	11	11	3.7	3.7
3	18	29	6.0	9.7
4	29	58	9.7	19.3
5	56	114	18.7	38.0
6	41	155	13.7	51.7
7	53	208	17.7	69.3
8	35	243	11.7	81.0
9	24	267	8.0	89.0
10	9	276	3.0	92.0
11	14	290	4.7	96.7
12	5	295	1.7	98.3
13	5	300	1.7	100.0
Total	300		100	

**Table 3:** Descriptive statistics of students’ total scores.

*N*	Minimum score	Maximum score	Mean	Std. deviation
300	2.00	13.00	6.51	2.43

Table 3 presents the descriptive statistics of the participants’ VHGT scores. The scores ranged from 2 to 13, with a mean of 6.51 (SD = 2.43). The distribution of scores provides descriptive information about students’ responses on the VHGT; however, the classification of students into Van Hiele levels was based on the specified level-classification criterion rather than on the mean score or a performance cut-off. The resulting student-level Van Hiele classifications are presented in Table 4.

**Table 4:** Van Hiele levels of geometric reasoning attained by students.

Levels	No Level reached	Level 0 (Visualization)	Level 1 (Analysis)	Level 2 (Informal deduction)	Level 3 (Deduction)	Total
*n*	67	99	95	31	8	300
*%*	22.3	33.0	31.7	10.3	2.7	100

Van Hiele level classification was based on responses to the adapted 20-item Van Hiele Geometry Test. “No identifiable level” refers to students whose response patterns did not satisfy the criteria for assignment to Levels 0–3.

Finally, presented in Table 4 are the student-level Van Hiele classification assessed by the VHGT. The assignment of students to the respective levels (0 to 3) was based on the weighted scoring procedure, where a student is considered to attain a level provided he or she correctly answers at least three out of the five items in each level. Here, a student is considered to attain and be placed at level n not just by satisfying the criterion at level n, but also the preceding level (n-1) (Usiskin, 1982). Thus, levels must be achieved in order, not randomly. Accordingly, if a student meets the “3 out of 5” criteria for success on Level 0, 1, and 3 but fails at Level 2, instead of being placed at Level 3, the student is placed at Level 1. The reason being that it is the highest level the student reached without skipping preceding levels. In the same way, if the criteria for Level 3 are achieved by a student but fail at Levels 1 and 2, no level is assigned.

As shown in Table 4, 22.3% (67) of the participants failed to attain any levels of Van Hiele geometric reasoning. For a greater proportion of the sampled students (*N* = 99, 33.0%), Level 0 (visualization) was attained. This pattern suggests that these students’ demonstrated geometric reasoning was primarily characterized by recognition of shapes based on their holistic appearance. Likewise, only 95 students (31.7%) progressed to Level 1 (Analysis). This suggests that relatively few students demonstrated the ability to identify geometric properties of figures. While Level 2 (Informal Deduction) was reached by just 31 (10.3%) students, Level 3 (Deduction), considered a higher level, was achieved by only 8 (2.7%) students, suggesting that only 8 students successfully navigated through all the levels, meeting the “3 out of 5” criteria.

Revealed in Table 4 is a clear trend, which has to do with a decline in the number of students as the level increases. In particular, the drop in the number of students from Level 1 to Levels 2 and 3 suggests that relatively few students in the study demonstrated progression from descriptive understanding to relational and, subsequently, formal deductive reasoning. This pattern suggests that, in terms of students’ geometric conceptual development, there is more room for improvement.

### 4.3 Students’ geometric problem-solving errors across Van Hiele levels

The analysis in this section focuses on the errors identified in students’ written responses to the geometric problem-solving test (GPST). Unlike the student-level Van Hiele classification reported in Section 4.2, the Van Hiele level considered here was determined at the level of each erroneous response. This response-level classification was used to identify the highest level of geometric reasoning explicitly evidenced in the written work for that particular response; it was not intended to represent a student’s underlying or general Van Hiele reasoning level. Thus, the “No identifiable level” category reported for some students in the VHGT classification in Section 4.2 does not constitute a category in the response-level analysis presented in this section. Students with no identifiable VHGT level were not excluded on that basis; instead, any erroneous GPST responses they provided were coded according to the highest Van Hiele reasoning level demonstrated in those individual responses. Accordingly, the Van Hiele level demonstrated in one GPST response was not assumed to apply to the student’s other responses. Differences in response-level classifications across items were therefore treated as differences in the reasoning evidenced in the written responses, not as changes in students’ underlying cognitive or developmental Van Hiele levels. This distinction is important because the response-based coding procedure was designed to capture the reasoning explicitly demonstrated within each response rather than to reassess students’ general Van Hiele levels across individual GPST items. Accordingly, each erroneous response was examined in relation to the highest Van Hiele level demonstrated in that particular response, after which the type of Newman error evident in the response was coded. The unit of analysis for this part of the study was therefore the erroneous response, rather than the individual students.

The error frequencies presented below therefore describe the distribution of coded erroneous responses and should not be interpreted as the number or percentage of students who exhibited each error type. Importantly, the Van Hiele levels shown in Table 5 are response-level classifications and are not the student-level VHGT classifications reported in Section 4.2. Thus, the eight students classified as Level 3 in the VHGT assessment should not be compared directly with the 454 erroneous GPST responses coded as demonstrating Level 3 reasoning. The former represents students classified once using the VHGT, whereas the latter represents individual erroneous responses that were independently coded according to the highest Van Hiele reasoning level demonstrated in each response. Across the 12-item geometric problem-solving test, 2,168 erroneous responses were identified and coded. Correct responses and items that were not attempted were excluded from this error analysis. The resulting dataset comprised repeated observations from the 300 students, since a student could contribute more than one erroneous response across the test items. The error frequencies presented below therefore describe the distribution of coded erroneous responses and should not be interpreted as the number or percentage of students who exhibited each error type. Table 5 presents the distribution of these errors according to the Van Hiele level demonstrated in the corresponding erroneous response.

**Table 5:** Errors distribution across demonstrated geometric reasoning levels.

Levels	Error types					Total	% of total errors
	RE (*n*)	CE (*n*)	TE (*n*)	PSE (*n*)	EE (*n*)		
0	32	142	76	32	197	479	22.09%
1	39	219	253	65	119	695	32.06%
2	27	137	208	108	60	540	24.91%
3	35	78	90	212	39	454	20.94%
Total	133	576	627	417	415	2,168	100%

The frequencies are presented descriptively; differences in raw counts across Van Hiele levels should not be interpreted as differences in propensity for a particular error category because the number of coded responses contributing to each level differs.

Of the total coded erroneous responses, TE (627, 28.92%) were the most frequent, followed by CE (576, 26.57%). PSE (417, 19.23%) and EE (415, 19.14%) were also represented, while RE (133, 6.13%) constituted the smallest proportion of the coded erroneous responses. This distribution shows that the coded errors were not evenly represented across the five Newman categories. The distribution of error categories also differed descriptively across the demonstrated Van Hiele levels. At Level 0, EE represented the largest category, with 197 of the 479 coded erroneous responses, followed by CE with 142 responses. At Level 1, TE accounted for 253 of the 695 coded erroneous responses, followed by CE with 219 responses. A similar descriptive pattern was observed at Level 2, where TE accounted for 208 of the 540 responses, while CE accounted for 137 responses. At Level 3, PSE represented the largest category, with 212 of the 454 coded erroneous responses, followed by TE (90) and CE (78). EE accounted for 39 responses at this level, while RE remained comparatively less frequent across the four demonstrated levels, ranging from 27 responses at Level 2 to 39 responses at Level 1. These frequencies describe the composition of the coded erroneous responses within each demonstrated Van Hiele level. They do not, by themselves, indicate that responses at one level were more likely to contain a particular error category because the number of coded erroneous responses differed across levels. Whether the observed differences in error-category representation were statistically associated with demonstrated Van Hiele level was examined using the category-specific GEE models presented in Section 4.4.

### 4.4 Association between Newman error categories and demonstrated Van Hiele levels

The association between Newman error categories and the demonstrated Van Hiele levels was examined using category-specific binary logistic Generalized Estimating Equation (GEE) models. GEE was used because the dataset contained multiple erroneous responses from the same students. Consequently, the 2,168 coded erroneous responses could not be treated as independent observations. StudentID was specified as the subject or clustering variable, while GPST Item was included both as a categorical explanatory factor to account for differences among the test items, including differences in content and cognitive demand, and as the within-subject repeated-measures factor to account for the repeated responses contributed by students across items. An exchangeable working correlation structure was applied to account for the dependence among responses contributed by the same student. Five separate GEE models were fitted, with each model examining one Newman error category. In each model, the selected error category was coded as 1, whereas all other Newman error categories were coded as 0. For example, in the CE model, a response classified as a CE was coded as 1, while responses classified as RE, TE, PSE, or EE were coded as 0. The demonstrated Van Hiele level was entered as a categorical explanatory variable, with Level 3 specified as the reference category.

This modeling approach examined whether the odds of a particular Newman error category relative to all other error categories differed across the demonstrated Van Hiele levels, after accounting for differences among GPST items and the correlation of repeated responses within students. The odds estimated by these models were conditional on a response being classified as erroneous. Thus, they represent the relative odds that an erroneous response belonged to the specified Newman error category rather than another Newman error category; they do not represent the probability that a student made that error across all GPST responses. The analysis was therefore restricted to the 2,168 coded erroneous responses, while correct and unattempted responses were excluded. The omnibus Wald test was used to examine whether the demonstrated Van Hiele level, considered as a set of categories, was associated with the relative odds of each Newman error category. Because Level 3 was the reference category, the omnibus test jointly examined the comparisons between Level 0 and Level 3, Level 1 and Level 3, and Level 2 and Level 3. A statistically significant omnibus Wald test indicates that at least one of these comparisons differs from the reference category. However, the omnibus test does not establish which specific comparisons are statistically significant. The individual odds ratios (ORs) and 95% confidence intervals (CIs) were therefore examined to identify the specific differences. Holm adjustment was applied to the five omnibus *p*-values to account for multiple testing. Table 6 presents the omnibus Wald tests for the five category-specific GEE models.

**Table 6:** GEE omnibus tests for the association between Newman error categories and demonstrated Van Hiele levels.

Newman error category	*n* (%)	Wald *χ*^2^	df	Holm-adjusted *p*
RE	133 (6.1)	3.918	3	0.270
CE	576 (26.6)	28.940	3	<0.001
TE	627 (28.9)	93.724	3	<0.001
PSE	417 (19.2)	306.854	3	<0.001
EE	415 (19.1)	198.134	3	<0.001

Each model compared the specified Newman error category with all other error categories. Student ID was treated as the clustering variable, and GPST Item was included as a categorical explanatory factor and as the within-subject repeated-measures factor. The percentages are based on the 2,168 coded erroneous responses.

The omnibus results, presented in Table 6, indicated that demonstrated Van Hiele level was statistically associated with the relative representation of CE [Wald *χ*^2^(3) = 28.940, Holm-adjusted *p* < 0.001], TE [Wald *χ*^2^(3) = 93.724, Holm-adjusted *p* < 0.001], PSE [Wald *χ*^2^(3) = 306.854, Holm-adjusted *p* < 0.001], and EE [Wald *χ*^2^(3) = 198.134, Holm-adjusted *p* < 0.001]. In contrast, the omnibus test for RE was not statistically significant [Wald *χ*^2^(3) = 3.918, Holm-adjusted *p* = 0.270]. These findings indicate that the demonstrated Van Hiele level was associated with the relative representation of four Newman error categories among the coded erroneous responses, after accounting for differences among GPST items and repeated responses within students. However, the significant omnibus results do not mean that every comparison between the lower Van Hiele levels and Level 3 was statistically significant. The specific comparisons for each error category are examined using the odds ratios and confidence intervals presented in Table 7.

**Table 7:** GEE odds ratios for Newman error categories by demonstrated Van Hiele level.

Newman error category	Level 0 vs. Level 3 OR (95% CI)	Level 1 vs. Level 3 OR (95% CI)	Level 2 vs. Level 3 OR (95% CI)
RE	0.857 (0.512–1.434)	0.711 (0.449–1.126)	0.631 (0.383–1.041)
CE	2.028 (1.460–2.817)	2.217 (1.639–2.994)	1.637 (1.180–2.273)
TE	0.760 (0.525–1.100)	2.309 (1.742–3.058)	2.532 (1.897–3.378)
PSE	0.082 (0.057–0.117)	0.118 (0.086–0.161)	0.285 (0.217–0.375)
EE	7.353 (5.128–10.526)	2.193 (1.504–3.195)	1.325 (0.848–2.070)

Level 3 was the reference category. Each odds ratio represents the relative odds, among erroneous responses, of the specified Newman error category relative to all other Newman error categories at the indicated Van Hiele level, compared with the corresponding odds at Level 3, after accounting for GPST Item and repeated responses within students. An OR greater than 1 indicates higher relative odds, whereas an OR below 1 indicates lower relative odds. A 95% CI that excludes 1 indicates a statistically significant comparison at the 5% level.

From Table 7, relative to Level 3, the relative odds of RE compared with all other error categories were lower at Level 0 (OR = 0.857, 95% CI [0.512, 1.434]), Level 1 (OR = 0.711, 95% CI [0.449, 1.126]), and Level 2 (OR = 0.631, 95% CI [0.383, 1.041]). However, all three confidence intervals included 1. This means that none of these pairwise comparisons was statistically significant. Thus, the analysis did not provide sufficient statistical evidence of an association between demonstrated Van Hiele level and the relative representation of RE.

Contrarily, the relative odds of CE compared to all other error categories were higher at each of the three comparison levels than at Level 3. At Level 0, the relative odds were approximately twice those at Level 3 (OR = 2.028, 95% CI [1.460, 2.817]). At Level 1, the relative odds were approximately 2.22 times those at Level 3 (OR = 2.217, 95% CI [1.639, 2.994]). At Level 2, the relative odds were approximately 1.64 times those at Level 3 (OR = 1.637, 95% CI [1.180, 2.273]). None of the confidence intervals included 1, indicating that all three comparisons were statistically significant. Within the coded erroneous responses, CE were therefore more strongly represented relative to the other error categories at Levels 0, 1, and 2 than at Level 3. Whereas these findings indicate statistically significant differences in the relative representation of Comprehension Errors between Level 3 and each of the other three demonstrated Van Hiele levels, they do not indicate that students at these levels were two times more likely to make Comprehension Errors across all test responses.

Concerning TE, the pairwise comparisons differed across the Van Hiele levels. At Level 0, the relative odds of TE compared to all other error categories were lower than at Level 3 (OR = 0.760, 95% CI [0.525, 1.100]). However, the confidence interval included 1, so this comparison was not statistically significant. At Level 1, the relative odds were approximately 2.31 times those at Level 3 (OR = 2.309, 95% CI [1.742, 3.058]). At Level 2, the relative odds were approximately 2.53 times those at Level 3 (OR = 2.532, 95% CI [1.897, 3.378]). The confidence intervals for both comparisons excluded 1, indicating statistically significant differences. Thus, Transformation errors had higher relative odds at Levels 1 and 2 than at Level 3, whereas the Level 0 and Level 3 comparison was not statistically significant.

In the PSE model, all three comparison levels had relative odds ratios below 1. Compared with Level 3, the relative odds of PSE as compared to all other error categories were approximately lower at Level 0 (OR = 0.082, 95% CI [0.057, 0.117]), Level 1 (OR = 0.118, 95% CI [0.086, 0.161]), and at Level 2 (OR = 0.285, 95% CI [0.217, 0.375]). All three confidence intervals excluded 1, indicating that the comparisons were statistically significant. Within the coded erroneous responses, PSE had substantially higher relative representation at Level 3 than at Levels 0, 1, and 2. The corresponding findings suggest that PSE constituted a greater relative share of the coded erroneous responses demonstrating Level 3 reasoning than of those demonstrating Levels 0, 1, and 2.

Likewise, EE were higher at Level 0 and Level 1 than at Level 3, but the Level 2 comparison was not statistically significant. At Level 0, the relative odds of EE compared to all other error categories were approximately 7.35 times those at Level 3 (OR = 7.353, 95% CI [5.128, 10.526]). At Level 1, the relative odds were approximately 2.19 times those at Level 3 (OR = 2.193, 95% CI [1.504, 3.195]). The confidence intervals for both comparisons excluded 1, indicating statistically significant differences. At Level 2, the estimated relative odds were approximately 1.32 times those at Level 3 (OR = 1.325, 95% CI [0.848, 2.070]). However, the confidence interval included 1, so this comparison was not statistically significant. Therefore, the analysis provided statistical evidence of differences in the relative representation of EE between Levels 0 and 3 and between Levels 1 and 3, but not between Levels 2 and 3.

### 4.5 Discussion of results

The findings from the selected senior high school showed that most students were classified at the lower student-level Van Hiele categories, particularly Level 0 (visualization) and Level 1 (analysis). Only a small number were classified at Level 2 (informal deduction), while very few were classified at Level 3 (deduction). This distribution indicates that, within the study setting, the student-level VHGT classifications were concentrated at the lower levels of the assessed Van Hiele hierarchy. It does not, however, provide evidence of developmental progression because the study was cross-sectional. This finding agrees with earlier studies which reported that many secondary school students were classified at lower levels of geometric reasoning. For instance, Alex and Mammen (2012) found that a large proportion of students operated at the visualization level, with only a few reaching deductive reasoning. Similar findings were reported by Usiskin (1982), Hoffer (1983), and Asemani et al. (2017), where most students were concentrated at Levels 0 and 1, with relatively few at higher levels. The present result also supports the work of Baffoe and Mereku (2010), who observed that many students entered SHS with weak geometric reasoning foundations and experienced difficulties with more advanced geometric reasoning.

The response-level evidence provides a more specific perspective on the reasoning represented in students’ written work. Some responses demonstrated visual recognition or identification of geometric properties, whereas others evidenced relationships among properties and more deductive forms of reasoning. These patterns can be interpreted in relation to cognitive accounts of geometric reasoning, which emphasize the roles of visual representation, attention, and the coordination of visual and relational information in geometric problem solving (Duval, 1999; Jones, 1998; Žakelj and Klančar, 2022; Dillon, 2026; Hohol, 2019). However, the present study did not directly measure students’ visual imagery, attention, working memory, or other underlying cognitive processes. Therefore, the observed response patterns should not be interpreted as direct evidence that students relied on surface appearance or particular cognitive mechanisms. Although the number of responses declined across the demonstrated Van Hiele levels, this distribution should be interpreted descriptively rather than as independent evidence for the hierarchical nature of the model, since the classification procedure itself incorporated the hierarchical ordering of the Van Hiele levels.

In terms of geometric problem solving, the analysis focused on the types of errors represented in students’ written responses and the Van Hiele reasoning levels demonstrated in those responses. Previous research has reported differences in problem-solving processes associated with Van Hiele reasoning levels (Andira and Syam, 2022; Ramadhania et al., 2022). In the present study, however, the analysis was conducted at the response level. Thus, the findings indicate how Newman error categories were represented among responses evidencing different levels of geometric reasoning rather than demonstrating that students at one Van Hiele level generally experience particular types of difficulty. This response-level approach allowed the demonstrated Van Hiele reasoning level and the Newman error category represented in the same erroneous response to be examined jointly.

The response-level analysis of the 2,168 erroneous responses produced by the students in the selected school showed that TE were the most frequent, followed by CE, while RE were the least recorded. This pattern shows that, among the coded erroneous responses, transformation difficulties were more frequently represented than reading difficulties. This finding supports Newman’s (1977) view that errors in mathematics can be examined according to identifiable stages of the problem-solving process. This result is also consistent with previous studies that identified TE and CE as prominent among secondary school students. For example, Arhin and Hokor (2021), Haryanti et al. (2019), and Obeng et al. (2024) reported difficulties associated with translating mathematical problems into appropriate representations or solution processes. Obeng et al. (2024) particularly found transformation errors to be the most common among SHS students. Within the present response-level dataset, the greater representation of TE and CE indicates that difficulties associated with interpreting and transforming geometric problems were more frequently evident in erroneous written responses than reading errors. This finding describes the location of difficulty in the written responses and does not by itself establish the cognitive processes underlying those difficulties.

PSE were also prominent in the response-level data, particularly among responses demonstrating higher levels of geometric reasoning. This pattern indicates that erroneous responses may contain relatively advanced demonstrated geometric reasoning while also showing difficulties in carrying out a mathematical procedure. It should not, however, be interpreted as evidence that Level 3 reasoning produces or is caused by procedural weakness. This observation is consistent with previous research showing that mathematical problem solving can involve distinct representational and procedural demands. Mirna (2018) and Azkiana et al. (2025), for example, reported difficulties in implementing procedures even where students appeared to identify appropriate solution approaches. In cognitive terms, this distinction is important because geometric reasoning and procedural execution need not be treated as a single process. Research on mathematical problem solving has shown that visual–spatial representations, working memory, and other cognitive resources can contribute to problem solving in different ways (Duval, 1999; Žakelj and Klančar, 2022; Dillon, 2026). The present study did not directly measure these processes, but its response-level findings indicate that demonstrated geometric reasoning and procedural error may co-occur within the same written response.

EE were also relatively frequent, particularly among responses demonstrating lower levels of geometric reasoning. At the response level, these errors involved difficulties in expressing final answers using appropriate units, notation, or mathematical statements. Rather than interpreting these patterns as evidence of a particular underlying cognitive weakness, they indicate that the final communication of a solution can remain problematic even when other aspects of a response demonstrate geometric reasoning. This interpretation is compatible with Pólya’s (1945) emphasis on reviewing and communicating solutions, although the present data do not permit conclusions about the cognitive reasons for students’ encoding difficulties.

A major finding of the study was the association between the Van Hiele reasoning level demonstrated in an erroneous response and the type of Newman error recorded in that response. The category-specific GEE analysis showed statistically significant differences across demonstrated reasoning levels for CE, TE, PSE, and EE, whereas the omnibus test for RE was not statistically significant after Holm adjustment. These findings indicate that, conditional on a response being classified as erroneous, the relative representation of Newman error categories varied across responses evidencing different Van Hiele reasoning levels, after accounting for differences among GPST items and repeated responses within students. EE constituted the largest category among Level 0 responses, while CE and TE were prominent among Level 1 and Level 2 responses, and PSE had greater relative representation among Level 3 responses. These descriptive patterns should be understood as response-level distributions rather than as evidence that students at particular Van Hiele levels were more or less likely to make specific errors across the GPST as a whole.

This result is consistent with the conceptual position that geometric reasoning and problem solving are related (Jones, 2000; Hohol, 2019). In the present study, responses evidencing different levels of geometric reasoning showed different relative patterns of Newman error categories. For instance, responses evidencing lower levels of reasoning showed greater relative representation of some error categories, whereas responses evidencing Level 3 reasoning showed a greater relative representation of PSE. These associations may reflect the fact that geometric problem solving requires the coordination of multiple forms of mathematical activity, including representation, relational reasoning, procedural execution, and communication. Cognitive research on geometry similarly emphasizes the role of visual–spatial representations and their coordination with reasoning processes in mathematical problem solving (Duval, 1999; Dillon, 2026; Žakelj and Klančar, 2022). Nevertheless, because the present study did not directly assess these cognitive processes, such explanations remain theoretically plausible interpretations rather than mechanisms established by the data.

These patterns do not establish that one variable causes changes in the other, but they indicate that demonstrated reasoning level and error type can co-occur in different ways within students’ written problem-solving responses. In this sense, the error categories provide an observable description of where difficulty was manifested within an erroneous response, while the Van Hiele coding provides an observable description of the level of geometric reasoning explicitly demonstrated in that response. Their integration therefore offers a response-level way of examining the interaction between geometric reasoning and problem-solving difficulty without assuming that the two represent a single underlying cognitive process. This pattern is consistent with the integrative proposition of the GRaPSE framework, which brings demonstrated geometric reasoning level and problem-solving error stage into a common analytical structure. The present findings provide an initial empirical examination of this proposed relationship within the study context, but they should not be interpreted as validation of the framework. Further research across different samples, contexts, and geometric problem-solving tasks is needed to examine the robustness and explanatory utility of GRaPSE.

The findings also have implications for cognitive psychology because they illustrate how observable features of mathematical responses can be used to examine the relationship between geometric reasoning and different stages of problem-solving difficulty. Rather than treating a geometric error as a unitary outcome, the present response-level analysis distinguishes the demonstrated reasoning evidenced in a response from the stage at which the error was manifested. This distinction is relevant to cognitive accounts of geometry, where visual–spatial representation, attention, relational reasoning, procedural processing, and communication may contribute differently to problem solving (Žakelj and Klančar, 2022; Jones, 1998; Hohol, 2019; Dillon, 2026). The study therefore provides an initial empirical basis for considering geometric problem-solving errors as response-level indicators that may be examined alongside demonstrated reasoning, while recognizing that direct measurement of the underlying cognitive processes requires methods such as think-aloud protocols, interviews, process tracing, or experimental tasks.

Within the context of the selected school, the findings suggest that examining geometric problem-solving responses may benefit from considering both the level of reasoning demonstrated and the stage at which a problem-solving difficulty is manifested. This implication concerns the interpretation and diagnosis of written responses rather than evidence that particular instructional approaches cause changes in reasoning. Instruction may therefore attend to visual recognition, analysis of geometric properties, relational reasoning, and deductive justification while also addressing difficulties evident at different stages of problem solving. These implications should, however, be interpreted in light of the study’s single-school setting rather than as evidence of patterns among pre-tertiary students generally.

## 5 Conclusion

This study examined the geometric reasoning levels and geometric problem-solving errors of final-year students in a selected senior high school through the combined perspectives of the Van Hiele Model of Geometric Reasoning and the Newman Error Hierarchical Model. The findings showed that most students in the study were classified at the lower levels of geometric reasoning, particularly visualization and analysis, with only a small proportion reaching informal deduction and very few attaining formal deduction. Analysis of the erroneous GPST responses showed that TE emerged as the most frequent, followed by CE, while RE were the least recorded, indicating that, among the erroneous responses examined, comprehension and transformation errors constituted a larger proportion of the coded errors than reading errors.

The category-specific GEE analysis further showed that the type of Newman error recorded in an erroneous response was associated with the Van Hiele reasoning level demonstrated in that response for CE, TE, PSE, and EE but not for RE after Holm adjustment. The findings therefore indicate that different problem-solving error patterns were observed across demonstrated reasoning levels rather than suggesting a uniform pattern of errors at each level. The proposed GRaPSE framework integrates the level of geometric reasoning demonstrated in a problem-solving response with the stage of difficulty represented by the Newman error categories, providing a conceptual structure for examining these dimensions jointly. In the present study, the observed associations offer an initial empirical examination of this proposed integration, but do not constitute validation of the framework. Further research is needed to examine its applicability and empirical robustness across different contexts and geometric problem-solving tasks. It is also noteworthy that these findings provide evidence from the students in the selected school and should be interpreted within this study context rather than as representative of pre-tertiary students in Ghana generally.

### 5.1 Implications for research and classroom practice

The findings of this study carry important implications for both classroom instruction and future research in mathematics education. Since most students were found to be classified at the lower Van Hiele levels of geometric reasoning, geometry instruction at the pre-tertiary level should move beyond routine memorization of formulas and procedures toward teaching approaches that deliberately promote reasoning development. Teachers need to design learning experiences that help students progress gradually from recognizing shapes based on appearance to analyzing properties, establishing relationships among figures, and engaging in deductive justification. Activities such as guided discovery, geometric investigations, diagram interpretation, and proof-oriented discussions may support this progression.

The dominance of transformation and comprehension errors also suggests that students require stronger support in interpreting problem statements and translating geometric situations into appropriate mathematical representations. Classroom practice should therefore place greater emphasis on students explaining their thinking, drawing accurate diagrams, justifying solution steps, and reflecting on final answers rather than focusing only on obtaining correct numerical results. Teachers should also make deliberate use of error analysis as a diagnostic strategy to identify where students’ problem-solving processes break down and to provide targeted instructional support. The observed differences in error patterns across demonstrated Van Hiele reasoning levels further suggest that diagnostic feedback should attend not only to whether a response is incorrect but also to the level of reasoning demonstrated and the particular stage at which the difficulty occurs.

The findings also indicate that assessment in geometry should not be limited to final answers alone. Teachers and curriculum developers should consider assessment practices that capture students’ reasoning processes and the specific nature of their errors. This would provide a clearer basis for remediation and instructional planning. For research, the findings support the usefulness of integrating the Van Hiele model and the Newman Error Hierarchical Model in studying students’ geometry learning. Accordingly, future studies may extend this work by validating the GRaPSE framework across different school contexts, grade levels, and mathematical topics beyond geometry to examine its broader applicability.

### 5.2 Limitations and suggestions for future research

This study was limited to final-year students in one selected senior high school, which restricts the extent to which the findings can be generalized to other schools and educational settings. Although the sample provided useful evidence on students’ geometric reasoning and problem-solving errors within the study context, differences in school type, instructional practices, curriculum implementation, and student backgrounds may be associated with different patterns in other settings. The findings should therefore be interpreted in relation to the selected school rather than as representative of pre-tertiary students in Ghana as a whole. Future studies may involve multiple schools across different regions and school contexts to provide broader comparative evidence and examine the consistency of the observed patterns. A further limitation concerns the distribution of students across the student-level Van Hiele categories. Although the study included 300 students, relatively few students were classified at the higher levels of the VHGT, particularly Level 3, where only eight students were classified. This uneven distribution limits the extent to which the student-level distribution of higher-order geometric reasoning can be interpreted beyond the study sample. It also highlights the need for caution when comparing reasoning patterns involving higher Van Hiele levels. Future research with larger and more diverse samples may provide greater representation of students at the higher levels and allow these patterns to be examined more robustly.

The study also relied mainly on achievement tests and written responses for identifying students’ reasoning levels and problem-solving errors. While this allowed for systematic quantitative analysis, written scripts may not fully capture the thinking processes behind students’ responses, particularly where reasoning is not explicitly expressed. It is worthy also to note that the Newman errors were diagnosed from students’ written responses rather than through diagnostic interviews, as such, the classifications indicate errors evident in the written work but may not conclusively establish the underlying cognitive source of those errors. In addition, the Van Hiele levels used in the GPST analysis were not pre-assigned to individual tasks. Rather, the highest level of reasoning explicitly demonstrated was inferred independently from each student’s written response. Consequently, because the GPST items differed in the opportunities they provided for demonstrating higher-level reasoning, the classification in the present study reflects the reasoning actually evident in the response rather than an *a priori* Van Hiele level assigned to the task. This response-based approach may therefore fail to capture reasoning that students possessed but did not express in writing. Future research may combine written assessments with interviews, think-aloud protocols, or classroom observations to provide richer evidence of students’ reasoning processes and to examine the relationship between task demands and demonstrated reasoning. Another limitation relates to the response-level nature of the error analysis. The dataset contained multiple erroneous responses contributed by the same students across different GPST items, meaning that observations were nested within students rather than representing independent participants. Although category-specific GEE models were used to account for the within-student correlation, the analysis was based on erroneous responses and therefore does not estimate students’ overall probability of making each Newman error across all attempted responses. The response-level analysis also means that the frequencies reported for Van Hiele levels represent erroneous responses rather than numbers or proportions of students. Future studies may use multilevel or longitudinal designs to examine both student-level and response-level variation while retaining the hierarchical structure of the data.

The study further focused on geometry within the core mathematics curriculum, particularly Strand 3, Geometry Around Us. The findings may therefore not fully represent students’ reasoning and error patterns across other areas of mathematics such as algebra, trigonometry, or statistics. Future research may examine the proposed GRaPSE framework across different mathematical domains and educational contexts to determine whether similar associations between demonstrated reasoning and problem-solving errors are observed. Such studies would also provide opportunities to further develop and empirically examine the proposed framework rather than treating the present findings as confirmation of its validity. Finally, the study did not employ an intervention or longitudinal design and therefore could not examine how students’ reasoning or error patterns change over time or under particular instructional conditions. Future intervention and longitudinal studies could investigate how students’ demonstrated reasoning and problem-solving errors develop across instructional periods and whether specific instructional approaches are associated with changes in these patterns. Such research may provide additional evidence for the practical use and further empirical development of the proposed GRaPSE framework.

## Data Availability

The raw data supporting the conclusions of this article will be made available by the authors, without undue reservation.

## Footnotes

1
https://uedufy.com/tools/sample-size-calculator/
