# Supplementary material for: A Van Hiele–Newman model analysis of geometric reasoning and problem-solving errors among final-year students in a selected senior high school
Source: Front Psychol. 2026 Oct 12;17:1870785. doi: 10.3389/fpsyg.2026.1870785 (PMC13696416; doi:10.3389/fpsyg.2026.1870785)
Supplement: Supplementary file 1 [file Supplementary_file_1.docx]

**Appendix A (Problem-Solving Tasks)**

**Kindly answer all questions in the spaces provided in the question paper.**

**60 Minutes**

1. A carpenter in a village is constructing a wooden window for a house. He makes sure that the window has four equal sides and that each corner forms a right angle. Identify the shape of the window.

2. Mr Agyapong, on his way to his daughter’s graduation ceremony, saw a triangular flag raised on a pole in front of the entrance of the school. How many sides does the flag have?

3. Mrs Pataku, a newly posted teacher to one special primary school, notices that the blackboard to be used for teaching was an egg-like shape. Identify the geometrical shape.

4. In a contest organised by the school, each of the contestants was asked to pick a shape and describe it. Akwasi Atimbila pick a shape which has four sides with exactly one pair of opposite sides parallel. Draw the shape picked by Akwasi Atimbila and name it.

5. Mr Atampuri, a local weaver, produces traditional mats used in homes and community centres. One of his mats is woven so that all four sides are equal in length, but none of the corners form a right angle. Draw the shape of the mat and name the shape.

6. Mr Kwame is constructing a simple table for his workshop. The tabletop is shaped so that both pairs of opposite sides are parallel, and the diagonals bisect each other. Draw the tabletop shape and name it.

7. Mr Atumpan, in a village called Esiekyem, used a rhombus and a kite panel shape in designing a fenced area for his farm. Panel $MNOP$ is a rhombus, and panel $PQNO$ is a kite attached to it. If $\angle PMN=76^{\circ}$ and $\angle PQN=120^{\circ}$, calculate the size of $\angle QPO$.

8. In a diagram, $A,B, C$ are points on the circle with centre $O$, and $/PQ/$ is a tangent to the circle at $B$. Given that the radius of the circle is $7cm$ with $\angle CBQ=35^{\circ}$, calculate, correct to two decimal places, the length of the minor arc.

9. A team of artists is designing a decorative wall hanging made of sticks arranged in triangular shapes. Points $B,C,E$ and $F$ lie on the same straight line, in that order. Point $A$, the top of the hanging, is connected downward to $C$ and $B$ to form $\Delta ABC$, while point $D$ is connected to $C$ and $E$, forming $\Delta CDE$. Given that $\angle A$ and $\angle B$ in $\Delta ABC$are 73° and 54° respectively, and $BA//CD$ and $AC//DE$, find the value of angle $x$ at point $E$ formed between $/DE/$ and the straight line $/EF/$.

10. A circular water tank has centre $O$. Points $A$, $B$ and $C$ lie on the circumference such that $\angle AOC=2x^{\circ}$. Justify that $\angle ABC$ subtended by arc $AC$ at any point on the remaining circumference is $x^{\circ}$.

11. Mr Kwame, a skilled carpenter in a village, is constructing a wooden notice board for a newly built classroom. The board is rectangular in shape *ABCD*. To ensure the frame is properly balanced, he measures the diagonals $/AC/$ and $/BD/$, which intersect at point $O$. Show mathematically that $/AO/= /CO/$ and $/BO/= /DO/$

12. During the annual durbar at a senior high school in Kumasi, the students are setting up a **triangular banner frame** for the chief guest. The frame is in the shape of triangle $PQR$, where the two sides $/PQ/$ and $/PR/$ are equal. A point $S$ is marked on $/PR/$ so that $/QS/=/QR/$. If the angle at $Q$ between $/PQ/$ and $/QS/$ is 30$^{\circ}$, **prove that** $\boldsymbol{\angle QPR}$ **is 10**$\boldsymbol{^{\circ}}$ **more than** $\boldsymbol{\angle PQS}$

**Appendix B**

**Blueprint Linking Each GPST Item to Curriculum Objectives and Cognitive Demands**

| **Item** | **Main geometry topic** | **Main curriculum objective** | **Curriculum competency assessed** | **Cognitive demand** | **Marks** |
| --- | --- | --- | --- | --- | --- |
| 1 | Quadrilaterals | Identify plane shapes | Identify common plane shapes from their defining characteristics | Understanding and classification | 2 |
| 2 | Triangles | Recognise properties of polygons | Recognise properties of triangles | Understanding and classification | 2 |
| 3 | Plane figures | Identify geometric figures | Identify two-dimensional geometric figures | Understanding and identification | 2 |
| 4 | Trapezium | Represent quadrilaterals | Draw and classify quadrilaterals | Applying and representation | 3 |
| 5 | Rhombus | Represent quadrilaterals | Identify and represent quadrilaterals | Applying and representation | 3 |
| 6 | Parallelogram | Properties of parallelograms | Recognise properties of parallelograms | Applying and analysing | 3 |
| 7 | Properties of quadrilaterals | Apply angle properties | Apply angle properties to solve problems | Applying and analysing | 7 |
| 8 | Circle theorem | Circle theorem and arc length | Apply circle properties in problem solving | Applying and analysing | 7 |
| 9 | Parallel lines and angles | Parallel lines and triangles | Solve problems involving angle relationships | Applying and analysing | 8 |
| 10 | Circle theorem | Angle at centre theorem | Justify geometric relationships | Analysing and justifying | 8 |
| 11 | Rectangle | Properties of rectangle diagonals | Justify properties of diagonals | Analysing and proving | 7 |
| 12 | Geometric proof | Geometric proof | Construct deductive geometric arguments | Analysing and proving | 8 |
| Total |  |  |  |  | 60 |

**Appendix C**

**Response-level coding rubric for classifying students’ demonstrated Van Hiele reasoning**

| **Van Hiele level** | **Operational coding criterion** | **Illustrative indicators in students’ written solutions** |
| --- | --- | --- |
| Level 0 (Visualisation) | The response recognises or identifies a figure primarily from its visual appearance without referring to defining properties or relationships. | Names or sketches a figure based mainly on its appearance; provides limited or no reference to geometric properties. |
| Level 1 (Analysis) | The response identifies one or more properties of a geometric figure but does not establish relationships among those properties. | Mentions equal sides, parallel lines, equal angles, diagonals, etc., without connecting them to justify a conclusion. |
| Level 2 (Informal deduction) | The response establishes relationships among geometric properties and uses informal logical reasoning to support a solution. | Links properties, explains why a property or theorem applies, or uses geometric relationships to develop a solution strategy. |
| Level 3 (Deduction) | The response presents a logically connected geometric argument based on accepted theorems or definitions or other established geometric principles | Sequential deductive proof, formal justification, or logically coherent mathematical argument. |
| Level 4 (Rigour) | The response demonstrates reasoning within formal axiomatic systems or compares alternative geometric systems. | Uses formal axiomatic reasoning extending beyond the senior high school curriculum. |

***Note.*** *Coding was conducted at the response level rather than the student or item level. Each written response was examined separately, and the highest level of geometric reasoning demonstrated by the reasoning evident in that response was recorded. Consequently, different responses from the same student could be classified at different Van Hiele levels***.**

**Appendix D. Response-Level Coding Procedure**

| **Coding stage** | **Procedure** | **Resulting information** |
| --- | --- | --- |
| Identify response | Each student’s GPST response was identified using the student’s unique identification code and the corresponding GPST item number. | Student ID and Item |
| Examine written reasoning | The complete written response was examined for the geometric reasoning demonstrated in the solution, rather than assigning a Van Hiele level based on the item itself. | Evidence of demonstrated reasoning |
| Assign Van Hiele level | The response was compared with the operational criteria in Appendix C, and the highest level of geometric reasoning demonstrated in that response was recorded. | VH Level (L0–L4) |
| Identify Newman error | Where an attempted response was incorrect, the response was examined using the Newman Error Hierarchical Model. | Newman Error |
| Link the two classifications | The Newman error identified in an erroneous response was linked to the Van Hiele level demonstrated in that same response. | VH Level × Newman Error |

***Note.*** *The response-level dataset used for the error analysis excluded correct and unattempted responses. A student could therefore contribute multiple observations and could have different demonstrated Van Hiele levels and Newman error categories across GPST items.*

**Appendix E. Coding Rubric Used for Classifying Newman Problem-Solving Errors**

| **Newman error category** | **Operational coding criterion** | **Typical indicators in students’ written solutions** | **Illustrative example** |
| --- | --- | --- | --- |
| Reading Error (RE) | The response shows evidence that the student misread or could not recognise relevant words, symbols, quantities, or mathematical information presented in the problem. | Misreads a numerical value, symbol, mathematical term, or other relevant information in the question. | The problem gives an angle as 73°, but the student uses 37° in the solution because the given value was misread. |
| Comprehension Error (CE) | The response shows that the student recognises the information in the problem but does not interpret its meaning or the mathematical requirement correctly. | Misinterprets what is being asked or misunderstands the meaning or relationship among the information provided. | The student identifies the given angles correctly but calculates an angle different from the one requested because the problem requirement was misunderstood. |
| Transformation Error (TE) | The response shows that the student understands the problem but selects an inappropriate mathematical operation, formula, theorem, or strategy to transform the given information into a solution. | Uses an inappropriate theorem, formula, operation, or solution strategy despite understanding the relevant information. | The student recognises the relevant angles but applies the angle-sum property of a triangle when the problem requires use of the angle at the circumference theorem. |
| Process Skills Error (PSE) | The response shows that the student selects an appropriate solution strategy but makes an error while carrying out the required mathematical procedures or calculations. | Incorrect substitution, arithmetic, algebraic manipulation, simplification, or execution of an otherwise appropriate procedure. | The student selects the correct formula and substitutes the given values correctly but makes an arithmetic error during calculation. |
| Encoding Error (EE) | The response shows that the student completes the relevant reasoning or procedure but records the final answer incorrectly, incompletely, or using inappropriate notation or units. | Incorrectly states the final answer, omits required units, or uses inappropriate mathematical notation despite otherwise appropriate reasoning. | The student obtains the correct length but records the final answer without the required unit or writes an incorrect final value despite correct preceding work. |
